# Humoral immunity and B-cell memory in response to SARS-CoV-2 infection and vaccination

**DOI:** 10.1042/BST20220415

**Published:** 2022-11-24

**Authors:** Holly A. Fryer, Gemma E. Hartley, Emily S.J. Edwards, Robyn E. O'Hehir, Menno C. van Zelm

**Affiliations:** 1 Allergy and Clinical Immunology Laboratory, Department of Immunology and Pathology, Central Clinical School, Monash University, Melbourne, VIC, Australia; 2 Allergy, Asthma and Clinical Immunology Service, Alfred Hospital, Melbourne, VIC, Australia

**Keywords:** COVID-19, IgG, memory B cell, neutralizing antibody, Omicron, SARS-CoV-2

## Abstract

Natural infection with SARS-CoV-2 induces a robust circulating memory B cell (Bmem) population, which remains stable in number at least 8 months post-infection despite the contraction of antibody levels after 1 month. Multiple vaccines have been developed to combat the virus. These include two new formulations, mRNA and adenoviral vector vaccines, which have varying efficacy rates, potentially related to their distinct capacities to induce humoral immune responses. The mRNA vaccines BNT162b2 (Pfizer-BioNTech) and mRNA-1273 (Moderna) elicit significantly higher serum IgG and neutralizing antibody levels than the adenoviral vector ChAdOx1 (AstraZeneca) and Ad26.COV2.S (Janssen) vaccines. However, all vaccines induce Spike- and RBD-specific Bmem, which are vital in providing long-lasting protection in the form of rapid recall responses to subsequent infections. Past and current SARS-CoV-2 variants of concern (VoC) have shown the capacity to escape antibody neutralization to varying degrees. A booster dose with an mRNA vaccine following primary vaccination restores antibody levels and improves the capacity of these antibodies and Bmem to bind viral variants, including the current VoC Omicron. Future experimental research will be essential to evaluate the durability of protection against VoC provided by each vaccine and to identify immune markers of protection to enable prognostication of people who are at risk of severe complications from COVID-19.

## Introduction

**Figure 1.**
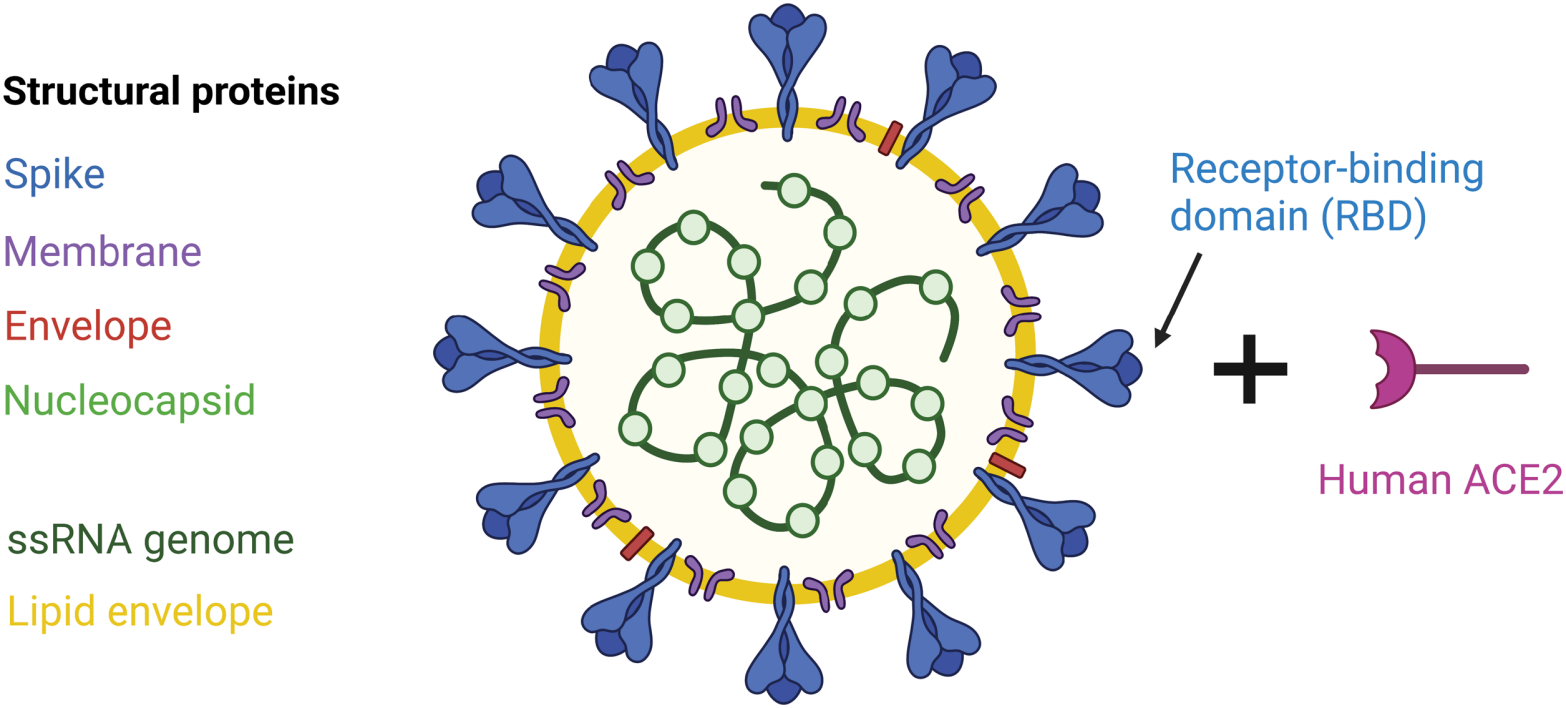
SARS-CoV-2 structural components and binding to human ACE2 receptor via the Spike RBD. The SARS-CoV-2 virus is composed of four main structural proteins: the Spike, Membrane, Envelope and Nucleocapsid. It carries a single-stranded RNA genome, which is encapsulated by a lipid envelope. The receptor-binding domain of the Spike protein binds to the human ACE2 receptor, allowing entry into host cells. ACE2, angiotensin converting enzyme 2; RBD, receptor-binding domain; ssRNA, single-stranded RNA. Created with BioRender.com.

The coronavirus disease 2019 (COVID-19) pandemic, caused by severe acute respiratory syndrome coronavirus-2 (SARS-CoV-2), is an ongoing global health threat with over 628 million cases and 6.5 million deaths as of November 2022 [1]. Genomic analysis suggests SARS-CoV-2 was initially transmitted from bats to humans through an intermediate species in a wet market in Wuhan, China, in late 2019 [2,3]. SARS-CoV-2 is a positive single-stranded RNA virus with four structural components: the Spike, Nucleocapsid, Envelope, and Membrane proteins (Figure 1) [4]. Cell entry and viral fusion are mediated by the receptor binding domain (RBD) in the S1 subunit of the Spike protein, which binds human angiotensin converting enzyme 2 (ACE2) (Figure 1) [5,6].

Since the virus first spread to humans in December 2019, multiple variants have arisen including variants of concern (VoC) which are defined based on their capacity for increased transmissibility, infectivity, and disease severity, and decreased effectiveness of public health measures such as vaccination, or therapeutics (Table 1) [7,8]. Following initial emergence of the Alpha, Beta, Gamma, and Delta VoC, the current circulating VoC is Omicron. Omicron has multiple sublineages, of which the BA.1, BA.2, BA.4, and BA.5 are spreading globally. Omicron BA.5 is the dominant SARS-CoV-2 strain globally (as of November 2022), accounting for over 80% of sequences globally, having recently overtaken BA.2 [9,10].

**Table 1:** Current and previous SARS-CoV-2 VoC and their RBD mutations

WHO variant name	Dates designated VoC (as of October 2022) [11]	PANGO lineage code [11]		Mutations in RBD (AA #319 to 541) [12]
Alpha	December 18 2020 – March 9 2022	B.1.1.7		N501Y [13]
Beta	December 18 2020 – March 9 2022	B.1.351		K417N, E484K, and N501Y [14]
Gamma	January 11 2021 – March 9 2022	P.1		K417T, E484K, and N501Y [15]
Delta	May 11 2021 – June 7 2022	B.1.617.2		L452R, T478K [16]
Omicron	November 26 2021 – present	B.1.1.529	Sublineage BA.1	G339D, S371L, S373P, S375F, K417N, N440K, G446S, S477N, T478K, E484A, Q493R, G496S, Q498R, N501Y, Y505H [9,17–19]
			BA.2	G339D, S371F, S373P, S375F, T376A, D405N, R408S, K417N, N440K, S477N, T478K, E484A, Q493R, Q498R, N501Y, Y505H, and reversion to Wuhan G446 and G496 [17,18]
			BA.4/5	All BA.2 RBD mutations + L452R, F486V, and reversion to Wuhan Q493 [20]

PANGO, Phylogenetic Assignment of Named Global Outbreak lineage; VoC, variant of concern; WHO, World Health Organization; RBD, receptor-binding domain.

Vaccines against SARS-CoV-2 are needed to combat outbreaks and control the pandemic. There has been global uptake of mRNA and adenoviral vector COVID-19 vaccines, which protect against severe disease and hospitalization [21,22]. However, the ability of these vaccines to induce durable humoral immune memory still needs to be established. This is important in the context of emerging VoC, as effective vaccines can help prevent viral variants from escaping immune protection and causing breakthrough infections, which are especially concerning for patients with underlying health conditions.

## The B-cell response and humoral memory production

Within several days following a primary viral infection, relatively low-affinity antigen-specific antibodies are produced by short-lived plasmablasts [23,24]. Memory B cells (Bmem) and long-lived plasma cells (LLPCs) are generated later in the immune response, remaining in the body long after the initial infection producing high-affinity antibodies [25]. While LLPCs maintain serum antibody levels, Bmem are primed to rapidly differentiate and secrete antibody upon subsequent exposures to the virus, providing efficient protection against infection and disease.

**Figure 2.**
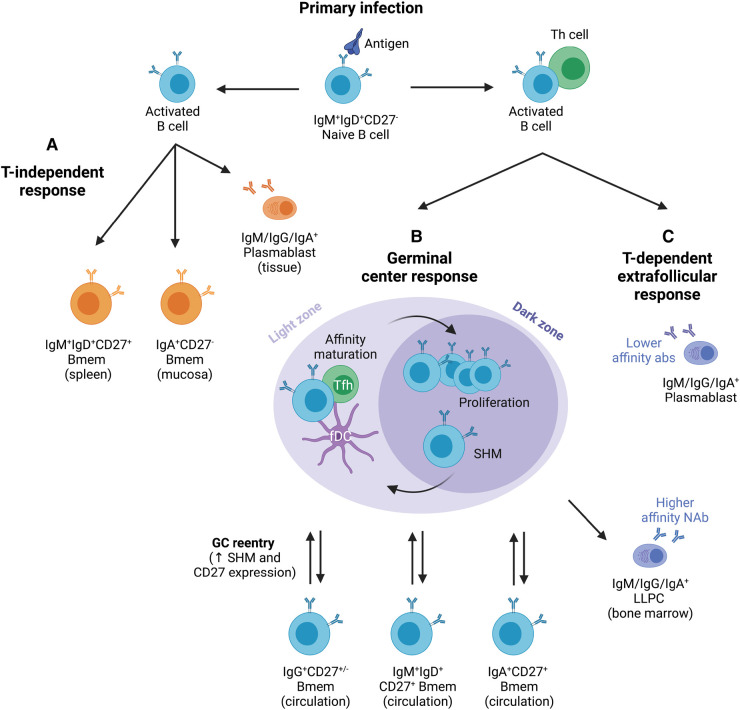
The different pathways of the humoral immune response generate plasmablasts, Bmem, and LLPC. Following activation, B cells follow either the T-independent or dependent pathways, depending on the structure of their cognate antigen. (**A**) The T-independent response generates plasmablasts, IgM^+^IgD^+^CD27^+^ and IgA^+^CD27^−^ Bmem. (**B**) The GC response is T-dependent and produces both LLPC and Bmem, which increase in CD27 expression and SHM levels upon re-entry. (**C**) The T-dependent extrafollicular response is GC-independent and generates short-lived plasmablasts which produce low-affinity antibody. Th, helper T cell; Tfh, follicular helper T cell; SHM, somatic hypermutation; GC, germinal center; fDC, follicular dendritic cell; LLPC, long-lived plasma cell. Based on information from [26,30,34–36]. Created with BioRender.com.

Bmem differentiate from activated B cells through two distinct pathways, either extrafollicularly or through the germinal center (GC) response in the B-cell follicles of secondary lymphoid organs (Figure 2) [26]. GC reactions are driven by the presence of cognate antigen, which is delivered to the draining lymph node via the subcapsular sinus or by dendritic cells migrating from the site of infection [27,28]. Follicular dendritic cells (fDCs) present antigen to GC B cells by capturing it mainly in the form of immune complexes [29]. B cells can endocytose soluble antigen or capture bound antigen from the surface of fDCs, which they then process and present to follicular helper T(fh) cells in the GC. These T-B cell interactions drive B-cell proliferation and expression of activation-induced cytidine deaminase (AID), inducing somatic hypermutation (SHM) of Ig variable domains and class-switch recombination (CSR) of Ig constant regions [30]. SHM increases the affinity of the surface Ig to recognize antigen presented by fDCs, such that B cells with a low affinity for antigen are outcompeted resulting in a pool of high affinity B cells [31,32]. CSR involves genomic rearrangement to switch from expressing the IgM isotype to one of four IgG subclasses (IgG1, 2, 3 and 4), one of two IgA subclasses (IgA1 and 2), or the IgE isotype. The various antibody isotypes are distributed throughout the body to exhibit distinct effector functions required for tailored protective immunity [33].

Recently activated B cells, which are antigen-experienced and proliferating, can be identified through the expression of CD71 [37]. Expression of CD20 distinguishes these cells from CD71^+^CD20^-^ plasma cells. The activation marker CD71 is down-regulated on activated B cells after 3–4 weeks as they transition into quiescent Bmem [37].

After exiting the GC, Bmem circulate and are readily detectable in peripheral blood. A large number of circulating Bmem express CD27, which is a tumor necrosis factor receptor for CD70 [38,39]. On Bmem expressing IgA or IgE, this marker distinguishes GC-independent Bmem (CD27^−^) from GC-derived Bmem (CD27^+^). On IgG^+^ Bmem, CD27 expression distinguishes cells derived from consecutive GC responses from the CD27^−^ primary responses [34]. The CD27^+^IgG^+^ Bmem typically have higher levels of SHM and are more frequently switched to the distal *IGHG* genes (encoding IgG2 and IgG4) than the CD27^−^IgG^+^ Bmem [34,40].

## The systemic humoral immune response to SARS-CoV-2 infection

Immediately following the SARS-CoV-2 outbreak, immunological studies were initiated to examine the serological and cellular responses post symptom onset (PSO), and their durability in convalescence. Following natural SARS-CoV-2 infection, antibodies specific for a number of viral epitopes have been detected. The RBD contains major neutralization epitopes, as antibodies that target these directly compete with ACE2 for binding, inhibiting viral fusion and cell entry (Figure 1) [41–46]. Most monoclonal antibodies isolated from Bmem that bound the RBD were found to be neutralizing, while those that bound epitopes on other SARS-CoV-2 proteins were mainly non-neutralizing [47]. A minority of human monoclonal antibodies specific for the neighboring N-terminal domain of the Spike protein are neutralizing, potentially acting by limiting the conformational changes needed for viral fusion [48]. The Nucleocapsid protein is another major antibody target, but being located inside the viral particle (Figure 1), it is not a neutralizing target [42,49]. Thus, the Spike RBD-specific immune response will be the focus of this review.

**Figure 3.**
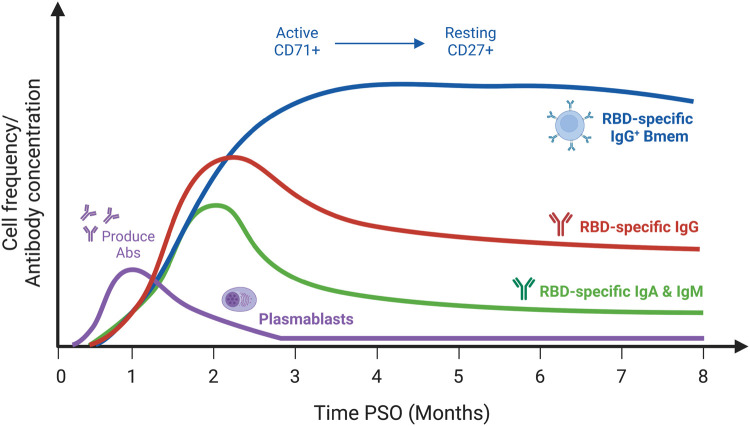
Kinetics of RBD-specific IgG, IgM, and IgA levels and plasmablast and Bmem numbers in SARS-CoV-2 convalescence. RBD-specific IgG levels decline following a peak at 2–4 weeks PSO but remain at detectable levels, whereas IgA and IgM levels drop more rapidly. Plasmablasts decrease after reaching peak levels ∼2 weeks PSO. RBD-specific Bmem numbers significantly increase in the months following infection, remaining at stable levels at least 6–8 months PSO. PSO, post-symptom onset; Bmem, memory B cells; Abs, antibodies; RBD, receptor-binding domain. Drawn in the style of Röltgen and Boyd [35], based on information from [41,43,45,50–52]. Created with BioRender.com.

In 95–100% of COVID-19 cases, SARS-CoV-2 Spike- and RBD-specific antibodies, predominantly of the IgG isotype, have been detected as early as the first week PSO [50–52]. RBD-specific antibodies peaked at 2–4 weeks PSO, followed by a significant decline (Figure 3) [41,44,45,50–53]. IgA and IgM levels decreased more rapidly than IgG, which remained detectable as late as 8 months PSO [41,43,52]. Neutralizing antibodies (NAb) against SARS-CoV-2 have been detected in 88% of COVID-19 cases, reaching peak levels at 1 month PSO followed by a decline [41,42,46,50,51,54]. An expansion of plasmablasts in peripheral blood following infection correlated with the peak in serum RBD-specific IgG [45,50,52]. Plasmablasts significantly decreased by 6 months PSO, signifying the resolution of acute infection and contraction of the antibody response (Figure 3) [44,55].

Up to 93% of SARS-CoV-2 convalescent individuals produced RBD-specific Bmem [41,56]. Numbers of Spike- and RBD-specific IgG^+^ Bmem increased over time and peaked ∼3–4 months PSO. In contrast with serum antibodies, Bmem numbers have not been shown to significantly decrease, remaining stable at 6–8 months PSO (Figure 3) [41,44,45,50,51,57]. Bmem isolated from infected individuals 3 months PSO produced RBD-specific antibodies with the capacity to potently neutralize viral entry [43,44,56]. Thus, natural infection elicits a Bmem population with the capacity to differentiate and secrete NAb to combat SARS-CoV-2 upon reinfection.

Between 1 and 6 months PSO, a shift from mainly activated, CD27^+^CD71^+^ Spike-specific B cells to mainly resting, CD27^+^CD71^−^CD21^+^ Bmem has been observed [44]. Other studies have also shown an increase in frequencies of CD21^+^CD27^+^ SARS-CoV-2-specific Bmem between 1 and 3 months PSO, indicative of GC-dependent B-cell formation [42,43]. Molecular analysis of Ig variable regions from RBD-specific Bmem revealed a gradual increase in SHM levels up to 6 months PSO [43,44,57]. This observation of extended affinity maturation indicates persistent GC activity driving Bmem formation, and could be the basis of the improved neutralizing capacity and breadth of antibodies cloned from SARS-CoV-2-specific Bmem at 6 months PSO [44,57]. These ongoing GC responses are potentially driven by persistence of SARS-CoV-2 antigens, which have been detected in the intestinal epithelium at least 4 months post-infection [57]. In conclusion, an Ig-class-switched, durable Bmem population is generated in response to SARS-CoV-2 infection, with the potential to provide long-term immunity.

## COVID-19 vaccine design

Vaccination triggers the capacity of the adaptive immune response to form antigen-specific immune memory by mimicking a primary pathogen exposure [58]. For SARS-CoV-2, the Spike protein was chosen as a major target, as antibodies against this surface protein have the capacity to neutralize the virus. The timing of the SARS-CoV-2 epidemic coincided with a new era in vaccine design, allowing induction of host cell production and presentation of Spike protein which ensures both humoral and cellular immune responses.

**Figure 4.**
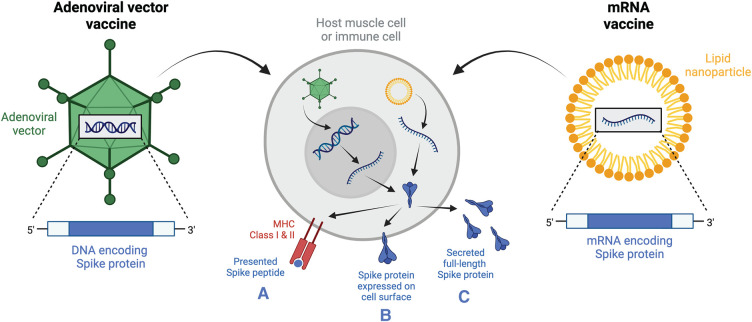
SARS-CoV2 mRNA and adenoviral vector vaccine designs. Adenoviral vector vaccines such as ChAdOx1 or Ad26.COV2.S comprise adenoviral vectors encapsulating the SARS-CoV-2 Spike protein DNA. When the vector is internalized into the host cell, the DNA is inserted into the nucleus where it is transcribed into mRNA before being translated into the Spike protein. mRNA vaccines such as BNT162b2 or mRNA-1273 comprise lipid nanoparticles containing Spike protein mRNA, which are internalized by recipient cells where the mRNA in is simply translated in the cytoplasm. The full-length Spike protein generated from either vaccine type can then be (**A**) processed and presented in MHC Classes I and II, (**B**) expressed on the cell surface, or (**C**) secreted from the cells, where it becomes a target for the immune system. MHC, major histocompatibility complex. Based on information from [62]. Created with BioRender.com.

The two new vaccine designs employed for SARS-CoV-2 are mRNA vaccines, BNT162b2 (Pfizer-BioNTech) and mRNA-1273 (Moderna), and adenoviral vector vaccines, predominantly ChAdOx1 (AstraZeneca) and Ad26.COV2.S (Janssen; Table 2). All vaccines are delivered in two-dose primary schedules except for Ad26.COV2.S, which is administered as a single dose. The mRNA vaccines both comprise mRNA transcripts for the proline-stabilized full-length Spike protein encapsulated in lipid nanoparticles (Figure 4) [59]. The mRNA is nucleoside-stabilized, meaning the RNA particles themselves are rendered non-immunogenic [60]. ChAdOx1 is a Y25 vector derived from a chimpanzee adenovirus, encapsulating dsDNA encoding the full-length Spike protein (Figure 4) [21]. Ad26.COV2.S has a similar design to ChAdOx1, except its Ad26 vector is derived from a human adenovirus, and the DNA encodes the proline-stabilized Spike protein form [61].

**Table 2:** Major COVID-19 vaccines and their effectiveness in protecting against symptomatic infection as reported by clinical trials

Vaccine name	Company	Vaccine type	# doses in primary schedule	Encoded antigen [63]	Efficacy against symptomatic infection	Efficacy against severe disease
Comirnaty (BNT162b2)	Pfizer-BioNTech	mRNA, lipid nanoparticle	2	Proline-stabilized full-length Spike protein	95% [22,64]	92% [65]
Spikevax (mRNA-1273)	Moderna	mRNA, lipid nanoparticle	2	Proline-stabilized full-length Spike protein	94% [66]	100% [66]
Vaxzevria (ChAdOx1)	Oxford-AstraZeneca	DNA, simian Y25 adenoviral vector	2	Wild-type full-length Spike protein	70.4% [67,68]	100% [69]
Jcovden (Ad26.COV2.S) [70]	Johnson & Johnson — Janssen	DNA, human Ad26 adenoviral vector [61]	1	Proline-stabilized full-length Spike protein	67% [71]	85% [71,72]

The mRNA and adenoviral vector vaccine particles are designed for rapid uptake by host cells, which use the RNA template for generation of Spike proteins. These proteins are processed in multiple ways to enable presentation to immune cells (Figure 4). There is evidence that the protein is presented on MHC Class I and II molecules, as both CD4^+^ and CD8^+^ T cells are generated in response to both vaccine types [62]. Additionally, host cells can express the full-length Spike protein on their surface or secrete it, where it becomes accessible to innate immune cells and B cells (Figure 4).

All four vaccines were demonstrated to be safe and effective at preventing symptomatic infection and hospitalization with COVID-19 in clinical trials, with a general tendency of mRNA vaccines to be slightly more effective than the adenoviral vector vaccines (Table 2). Following their roll-out, all vaccine formulations were found to greatly reduce the incidence of COVID-19 infection, severe disease outcomes, and death in the general population [73–76]. Immunizations with ChAdOx1 and Ad26.COV2.S have been linked to rare occurrences (1–2 per 100 000 vaccinees) of thrombosis with thrombocytopenia, resulting in hesitancy in their uptake and the recommendation to only use mRNA vaccines for booster immunizations [77–80].

## The antibody response elicited by mRNA and adenoviral vector COVID-19 vaccines

**Figure 5.**
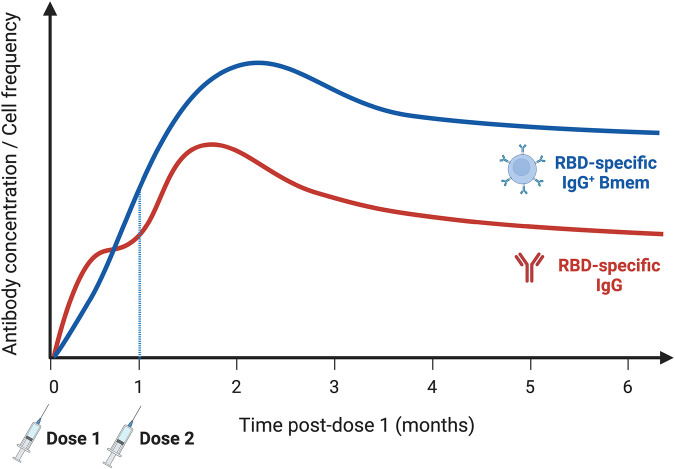
Kinetics of the antigen-specific antibody and Bmem response to mRNA vaccines up to 6 months post-vaccination. The Spike- and RBD-specific IgG response is boosted by each vaccine dose, and peaks 1 week post-dose two, before declining. RBD-specific Bmem increase in the months following vaccination and remain at stable levels at least 6 months post-dose one. Bmem are of a class-switched resting phenotype and increase in Ig affinity over time. RBD, receptor-binding domain; Bmem, memory B cells. Based on information from [82,83]. Created with BioRender.com.

Similar to natural infection, the COVID-19 vaccines BNT162b2, mRNA-1273, ChAdOx1, or Ad26.COV2.S predominantly elicit an IgG response [81–86]. After mRNA vaccination, Spike- and RBD-specific IgG and NAb have been found to peak within 1 month post-dose two (Figure 5), correlating with the peak of IgG^+^ plasmablasts [82,83,87,88]. Following a rapid decline in RBD-specific IgG and NAb levels, these then remained relatively stable between 3–6 months [82,83,87,88]. This decline in serum antibody levels correlated with a waning in protection against symptomatic infection [89]. However, the high level of mRNA vaccine protection from hospitalization and death has been shown to remain stable until at least 5 months post-dose two, suggesting that cellular immune memory rather than circulating antibody levels contribute to long-term protection against severe disease.

A single dose of ChAdOx1 or Ad26.COV2.S resulted in detectable serum NAb in 91–100% of recipients [21,90–92]. Spike-specific IgG peaked 2–4 weeks after one dose of ChAdOx1, followed by a contraction before the second dose, which boosted both IgG and NAb levels [21,84,93–95]. Similar to mRNA vaccination, RBD-specific IgG levels also declined between 1 and 6 months after Ad26.COV2.S vaccination, accompanied by a loss of detectable NAb in 39% of individuals by 6 months post-vaccination [96].

NAb and Spike-specific IgG levels elicited by both adenoviral vector vaccines, ChAcOx1 and Ad26.COV2.S, are significantly lower than those elicited by two doses of mRNA vaccines [85,93,95–98]. The diminished response to ChAdOx1 could be due to the lack of stabilizing proline mutations, which maintain the prefusion Spike conformation, which has been found to be more immunogenic [22,66,99–102].

## Bmem formation following mRNA and adenoviral vector COVID-19 vaccinations

After two doses of either mRNA vaccine, Spike-specific IgG^+^ Bmem have been detected in 90-100% of individuals [82,83]. The second dose boosted Bmem numbers and increased the frequency of high-affinity, isotype-switched Bmem [83]. Despite some variability in the detailed kinetics of Bmem after the second dose of an mRNA vaccine, Bmem frequencies measured 6 months after dose two stayed above post-dose one levels and were comparable to those formed after natural infection (Figure 5) [82,83]. Similarly, SARS-CoV-2-specific Bmem were detected in all individuals fully vaccinated with adenoviral vector vaccines [103]. In the 6 months following Ad26.COV2.S vaccination, RBD-specific Bmem increased in number; however, their absolute numbers and frequencies remained lower than those generated by mRNA vaccines [96]. While one dose of ChAdOx1 elicited detectable Spike-specific Bmem in only 53% of vaccinees, these numbers significantly expanded after the second dose and were detectable in all individuals [94]. Recipients of ChAdOx1 had lower frequencies of RBD-specific Bmem than mRNA vaccinees, but the affinity and neutralization activity of monoclonal antibodies isolated from these Bmem were similar between the two vaccine types [104].

One week after dose two of an mRNA vaccine, there was a reduction in the frequency of CD71^+^ Bmem, suggesting a shift to a resting memory population [82]. Following *in vitro* stimulation, these Spike-specific Bmem rapidly reactivated and produced neutralizing IgG, demonstrating their capacity to protect against secondary infections [82]. SARS-CoV-2-specific Tfh cells were produced after one mRNA vaccine dose, and their levels correlated with the neutralizing capacity of IgG [81,105]. This suggests that in response to mRNA vaccines, maturation of Bmem is supported by Tfh cells in GC reactions. Additionally, Spike-specific GC B cells were detected in draining lymph nodes as late as 3–4 months post-dose two, indicating sustained GC activity, similar to natural infection [44,87]. Further research is required into the phenotype, kinetics, and durability of the Bmem response generated by both adenoviral vector vaccines, to inform the extent of protective memory conferred by each vaccine.

## Vaccine-elicited humoral immune response effectiveness against SARS-CoV-2 VoC

While all four mRNA and adenoviral vector vaccines elicited strong antibody and Bmem responses against the parental Wuhan SARS-CoV-2 strain, NAb levels against previous and current VoC were reduced [15,106,107]. This reduction in reactivity to variants compared with Wuhan mostly resulted from mutations in the Spike RBD (Table 1) [108–110]. Continued close monitoring of vaccine effectiveness against emerging variants is essential, as viral escape from the immune response can lead to increases in severe disease incidence from breakthrough infections.

**Figure 6.**
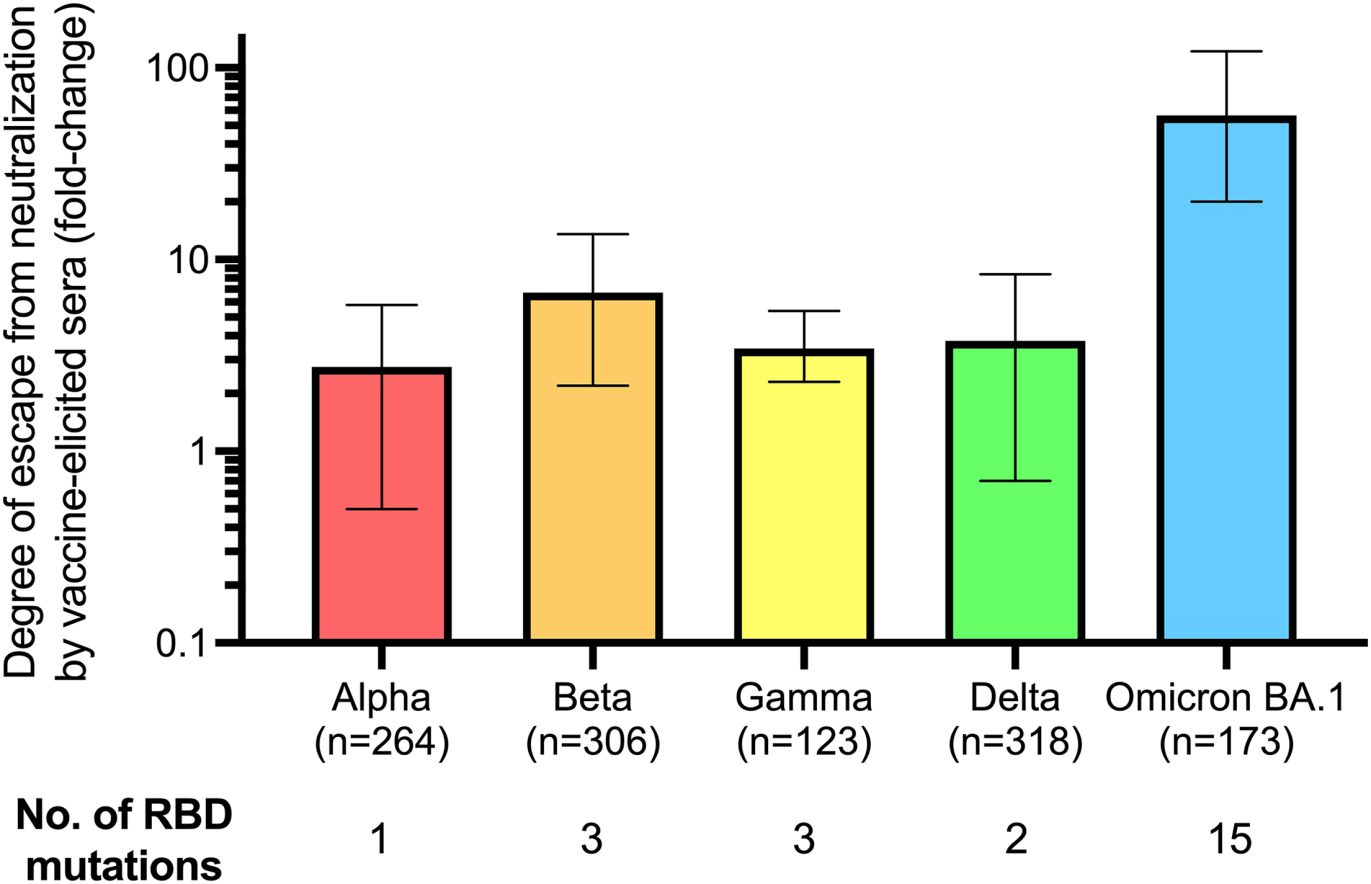
Relative escape of SARS-CoV-2 VoC from neutralization by sera from recipients of two mRNA vaccine doses. Each of the five main past and present VoC escape neutralization by vaccine-elicited sera to varying degrees. NAb titers against Alpha are, on average, 2.75 times lower compared with those against Wuhan [106,119–122], those against Beta are 6.74-fold [106,109,112,119,120,122,123], those against Gamma are 3.43-fold [109,120,124], those against Delta are 3.76-fold [106,119–123], and those against Omicron are 56.50-fold lower [107,119,123,125]. Values are presented as mean fold change compared with Wuhan, error bars represent the range of the referenced studies. Total number of serum samples from pooled publications indicated. NAb, neutralizing antibodies; RBD, receptor-binding domain; VoC, variants of concern.

Past VoC have evaded the humoral immune response elicited by vaccines to varying degrees (Figure 6). Following two doses of either BNT162b2 or ChAdOx1, most studies reported a 2–3-fold lower NAb titer against the Alpha variant than to the original Wuhan strain [109,111]. In contrast, NAb levels generated by BNT162b2 or ChAdOx1to the Beta variant were 6–7-fold lower than to Wuhan [96,106,107,112]. The escape from neutralization by these variants can be partially explained by RBD mutations that reduce recognition by NAb such as N501Y shared by Alpha, Beta, and Gamma, which has been linked to increased Spike affinity for ACE2, and K417N/T and E484K in Beta (Table 1) [107,109,110,113–115]. Despite having a similar RBD sequence to Beta except for the substitution at position 417 (K417T), NAb titers against the Gamma variant have been found to be only 2–6-fold lower than those against Wuhan after two doses of an mRNA vaccine [108,109,116]. The difference in NAb titers against Beta and Gamma can be attributed to differences in their Spike protein sequences outside of the RBD, including in the N-terminal domain which has been shown to contain NAb targets [117,118].

The Delta variant was the dominant strain globally in late 2021 before the emergence of Omicron [11]. The RBD mutations in Delta, L452R and T478K, increase its affinity for ACE2, while reducing serum antibody binding [109]. One dose of either BNT162b2 or ChAdOx1 failed to induce serum NAb titers against Delta in ∼90% of individuals [15,106]. A second dose improved NAb levels against Delta, although these remained 2.5–6-fold lower than against Wuhan [15,106,109] (Figure 6). A study in Singapore showed no correlation between NAb levels and the incidence of breakthrough Delta infection [126]. However, higher RBD-specific Bmem levels correlated with a reduced likelihood of being infected, suggesting these are an accurate marker of protection against VoC [126]. Six months after two mRNA vaccine doses, more than 50% of all RBD-specific Bmem recognized the Alpha, Beta, and Delta variants, demonstrating that the capacity of Bmem to bind VoC was higher than that of the NAb response [82].

The original Omicron strain, BA.1, carries 15 RBD mutations, including: N501Y, shared by all past VoC; K417N, shared with Beta; and T478K, shared with Delta (Table 1) [11,17,20,118]. The BA.2 sublineage has 16 RBD mutations, with six differences from the sequence of BA.1 [20,118]. BA.4 and BA.5 carry 17 RBD mutations, and only differ from each other outside the Spike protein. These two variants share all BA.2 RBD mutations except for Q493R, while having acquired L452R and F486V [20,118].

The emergence of Omicron subvariants has led to greater rates of reinfections, breakthrough infections in vaccinated individuals, and community transmission than Delta and other ancestral variants [127–129]. One suggested mechanism behind these increased infection rates is the dramatically increased capacity of Omicron to replicate in the bronchi [130,131]. However, Omicron is detected less in the lower lung parenchyma, which could explain the reduced disease severity from infections with this variant [130,131]. The increased transmissibility of Omicron can be linked to a reduction in the protection provided by primary schedule vaccination. All Omicron sublineages significantly escape neutralization in recipients of mRNA and adenoviral vector vaccines, likely due to the large number of mutations these variants carry in their Spike and RBD (Table 1) [17,108]. Multiple studies reported that most double-vaccinated individuals have no detectable Omicron-reactive NAb, with levels being 20–120-fold lower against BA.1 than Wuhan [108,119,123,125,132–134] (Figure 6). Both BA.4 and BA.5 escaped neutralization from serum antibodies of BNT162b2 and Ad26.COV2.S recipients to a higher degree than BA.1 [135–137].

A third mRNA vaccine dose, given to recipients of both mRNA and adenoviral vector primary vaccines, has been shown to increase protection against severe disease and death from SARS-CoV-2, including against Delta and Omicron [138–142]. This third dose booster may achieve protection in part by raising Spike- and RBD-specific IgG and NAb levels [116]. Notably, the third dose is reported to increase the neutralization capacity of serum antibody against BA.1 and BA.2 up to 100-fold, and against Delta up to 30-fold [108,119,125,143–145]. A third dose of BNT162b2 increased NAb against BA.4/5, but levels still remained at least 5-fold lower than against Wuhan [135–137]. These elevated serum NAb levels against VoC may be due to the Omicron-binding Bmem pool being reactivated by the third vaccine dose, inducing differentiation into antibody-secreting cells.

While the antibody response elicited by two doses of a COVID-19 vaccine is less effective against the Omicron sublineages, vaccine recipients still produce substantial frequencies of Bmem that recognize the VoC. A study using Spike and RBD probes to measure specific Bmem frequency by flow cytometry found similar frequencies of Bmem that bound Wuhan, Alpha, Delta, and Omicron BA.1 after two doses of BNT162b2, with a majority of Bmem being able to bind multiple variants [135]. A third dose or an infection with BA.1 increased variant-binding Bmem frequencies [135]. Another group found the proportion of resting Wuhan-specific IgG^+^ Bmem that bound Omicron BA.1 increased between 1- and 4–5-months post-dose two of BNT126b2 [146]. This demonstrates that Omicron-binding Bmem are generated following the primary dosing schedule of mRNA COVID-19 vaccines, mature over time, and are boosted by repeat exposures.

Higher levels of SHM were observed in the Ig genes of variant-binding Bmem than in those that only recognized the Wuhan strain [82,147]. Thus, the generation of higher-affinity Bmem through GC reactions may be advantageous for the recognition of VoC. This highlights a difference in the variant-binding capacity of the circulating antibody pool, produced by plasmablasts, and the resting Bmem population. A functional benefit that Bmem possess over terminally differentiated plasma cells, which have fixed specificity, is the capacity to evolve their antibody binding affinity and breadth [148,149]. This helps explain why COVID-19 vaccines elicit low plasma NAb levels against VoC, but higher frequencies of variant-specific Bmem. Similar Bmem analyses are still needed in the context of adenoviral vector vaccines, as well as further insights into the effectiveness of Bmem generated by either vaccine formulation against BA.2 and BA.4/5.

## Conclusion

Effective vaccines that generate immune memory against SARS-CoV-2 and emerging variants are required to provide durable protection against severe COVID-19. Spike-specific IgG and NAb levels wane following both infection and a primary schedule of vaccination, suggesting serum antibodies are not the most robust marker of protection. In contrast, Bmem numbers are more stable months after infection or mRNA vaccination, indicating that this cellular marker of immune memory may be a better correlate of protection against severe disease with SARS-CoV-2. The kinetics of the Bmem response elicited by ChAdOx1 or Ad26.COV2.S have not been studied in detail. This research will still be insightful as although many countries are now delivering only mRNA booster doses, primary courses of adenoviral vector vaccines have been widely administered around the world.

Vaccine-elicited antibodies have a reduced neutralizing capacity against past and current VoC. Nevertheless, a third mRNA vaccine dose elevates antibody levels and increases the capacity of these antibodies to recognize variants. Variant-binding Bmem are elicited by mRNA vaccines; however, equivalent studies have not yet been performed in the context of ChAdOx1 or Ad26.COV2.S vaccination. Additionally, both serological and cellular measures of the immune response against Omicron BA.2 and BA.4/5 generated by all vaccine types requires more research. This will provide insight into the capacity of the current COVID-19 vaccines to induce humoral immune memory capable of long-term protection against severe breakthrough infections with VoC.

## Perspectives

- Natural infection and mRNA COVID-19 vaccination both elicit IgG-dominated antibody responses of similar magnitude, and robust resting Bmem populations. Adenoviral vector vaccines elicit lower serological responses, but insights into the Bmem response to this vaccine type are limited. Booster mRNA vaccines induce a rise in serum antibody levels and an expansion of Bmem, as well as the capacity of these to recognize VoC, regardless of the primary vaccine formulation.
- While serum antibody levels induced by COVID-19 vaccines protect against infection, this effect rapidly wanes within 1–3 months. Vaccine-induced protection against severe disease lasts for at least 4–6 months and correlates better with the durability of immune memory cells than with the rapid contraction of the antibody response.
- Quantitative and qualitative measurements of COVID-19 vaccine-induced humoral immune memory may provide immunological markers for protection from severe disease in vaccinated individuals. Future research is also required to expand the evidence base for the level of protection current Wuhan-based vaccines elicit against Omicron variants, and the need to update the vaccine strains to protect from infections with VoC.

## Open Access

Open access for this article was enabled by the participation of Monash University in an all-inclusive *Read & Publish* agreement with Portland Press and the Biochemical Society under a transformative agreement with CAUL.

## Abbreviations

ACE2: angiotensin-converting enzyme 2
Bmem: memory B cell
COVID-19: Coronavirus disease 2019
CSR: class-switch recombination
fDC: follicular dendritic cell
GC: germinal center
LLPC: long-lived plasma cell
NAb: neutralizing antibody
PANGO: Phylogenetic Assignment of Named Global Outbreak lineage
PSO: post-symptom onset
RBD: receptor binding domain
SARS-CoV-2: severe acute respiratory syndrome coronavirus-2
SHM: somatic hypermutation
Tfh: follicular helper T cell
VoC: Variant of concern
WHO: World Health Organization
